# Supplementary figures and images for: Nonameric structures of the cytoplasmic domain of FlhA and SctV in the context of the full-length protein
Source: PLoS One. 2021 Jun 18;16(6):e0252800. doi: 10.1371/journal.pone.0252800 (PMC8213127; doi:10.1371/journal.pone.0252800)

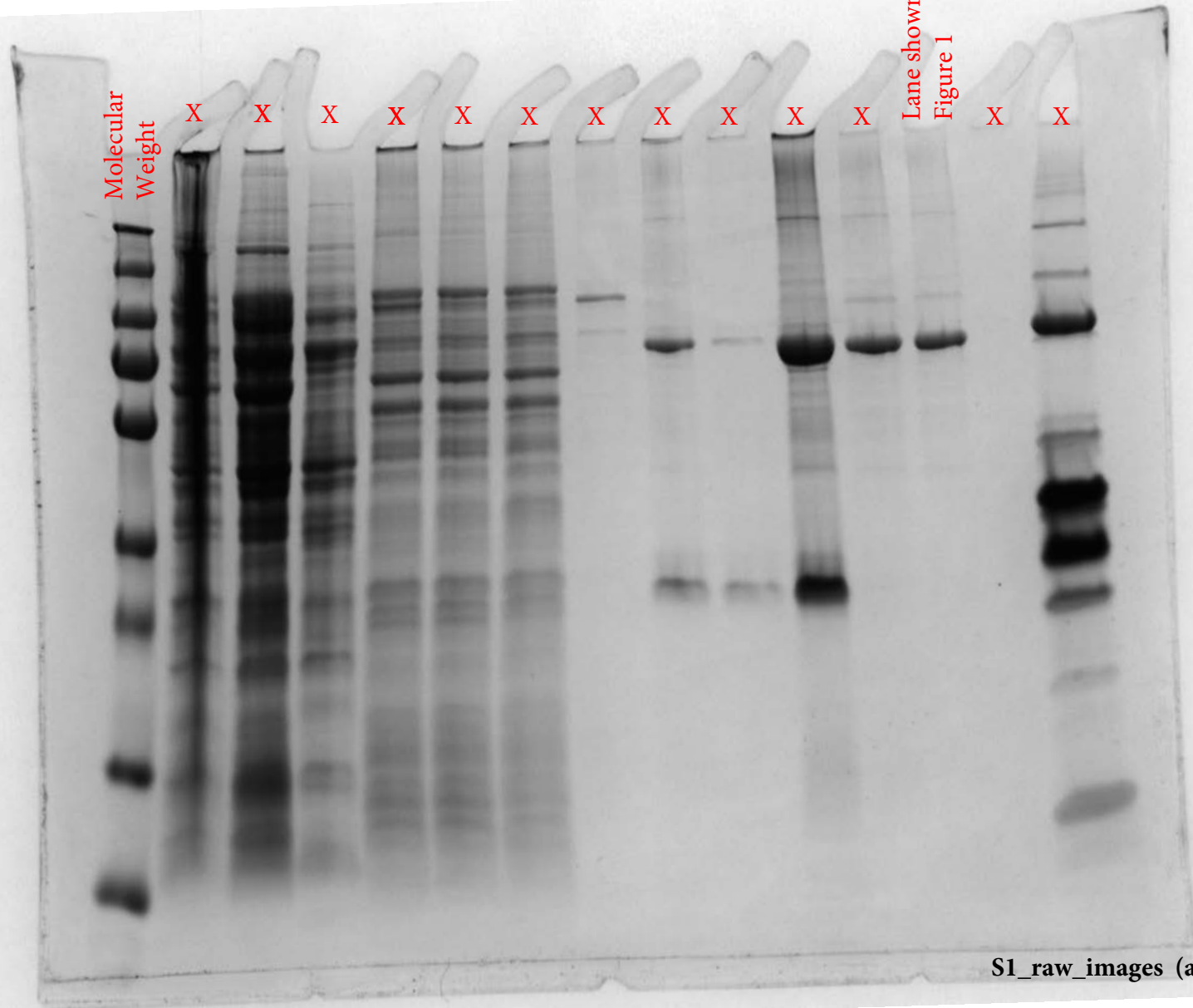

Molecular  
Weight

X

X

X

X

X

X

X

X

X

X

lane shown  
in figure 1

X

Supplement: S1 Raw images — Samples as described in Fig 1. Panel (a) shows the complete raw gel from which the lanes shown in Fig 1(B) left hand side are taken, panel (b) shows the complete raw gel from which the lanes shown in Fig 1(B) right hand side are taken. In both images, lanes marked X are either from different points during purification of those samples or from unrelated preparations. (PDF) [file pone.0252800.s001.pdf]
