# Supplementary material for: Nonameric structures of the cytoplasmic domain of FlhA and SctV in the context of the full-length protein
Source: PLoS One. 2021 Jun 18;16(6):e0252800. doi: 10.1371/journal.pone.0252800 (PMC8213127; doi:10.1371/journal.pone.0252800)
Supplement: Attachment — Submitted filename: r2r_ed.docx [file pone.0252800.s002.docx]

Response to reviewers

1. Please ensure that your manuscript meets PLOS ONE's style requirements, including those for file naming. The PLOS ONE style templates can be found at

https://journals.plos.org/plosone/s/file?id=wjVg/PLOSOne_formatting_sample_main_body.pdf and

[https://journals.plos.org/plosone/s/file?id=ba62/PLOSOne_formatting_sample_title_authors_affiliations.pdf](about:blank)

 Done

2. Thank you for stating the following in the Acknowledgments Section of your manuscript:

"The work was funded by grants from the Wellcome Trust 109136,
100298, 219477, the Medical Research Council (UK) S021264 and the Wolfson Foundation
WL160052. Work at the CSB was funded by core funding from the CCR."

We note that you have provided funding information that is not currently declared in your Funding Statement. However, funding information should not appear in the Acknowledgments section or other areas of your manuscript. We will only publish funding information present in the Funding Statement section of the online submission form.

Please remove any funding-related text from the manuscript and let us know how you would like to update your Funding Statement. Currently, your Funding Statement reads as follows:

"The funders had no role in study design, data collected and analysis, decision to publish, or preparation of the manuscript"

Please include your amended statements within your cover letter; we will change the online submission form on your behalf.

 Thank you – we have removed the text from the manuscript. The funding statement should read:

The work was funded by grants from the Wellcome Trust 109136, 100298, 219477, the Medical Research Council (UK) S021264 and the Wolfson Foundation WL160052. Work at the CSB was funded by core funding from the CCR. The funders had no role in study design, data collected and analysis, decision to publish, or preparation of the manuscript

3. We note you have included a table to which you do not refer in the text of your manuscript. Please ensure that you refer to Table 1 in your text; if accepted, production will need this reference to link the reader to the Table.

Apologies – we have included the table in this submission

Reviewers' comments:

We thank both reviewer’s for their thoughtful consideration of our manuscript. We address specific points below and hope the amended text is now suitable for publication in PLOS One.

Reviewer #1: Pathogenic bacteria utilize type III secretion system (T3SS) to directly inject virulence-associated effector proteins into the cytosol of host cells for their invasion. They also use the T3SS to construct the bacterial flagella on the cell surface. The protein export apparatus of the T3SS is composed of a transmembrane export gate complex powered by a proton motive force across the cell membrane and a cytoplasmic ATPase. FlhA in the flagella and SctV in virulence-associated T3SSs are thought to be an ion channel to couple the ion flow through the channel to protein translocation across a polypeptide channel of the export gate complex. Both C-terminal cytoplasmic domains of full-length FlhA (FlhAC) and SctV (SctVC) form homo-nonamer in the T3SS and mediate hierarchical protein targeting and secretion. In this study, Kuhlen et al. solved C-terminal cytoplasmic domains of full-length FlhA and SctV by cryoEM image analysis and demonstrated that FlhAL and SctVL bind to their neighboring subunits in the FlhAC and SctVC rings, respectively, not only to stabilize the nonameric ring assembly but also to adopt an open form. All experiments are carefully and well designed. Also, all data are clearly presented and well documented. This research article would be of interest to general readership, providing important advancements in our knowledge on the protein export mechanism of the T3SS. However, this reviewer has a few minor comments.

1. Introduction, the second paragraph: In addition to references #5 and 6, the author should also cite the following two papers:

Fukumura, T. et al. Assembly and stoichiometry of the core structure of the bacterial flagellar type III export gate complex. PLOS Biol. 15, e2002281 (2017).

Ward, E. et al. Type-III secretion pore formed by flagellar protein FliP. Mol. Microbiol. 107, 94–103 (2018).
References added as requested

2. P5, line 4-5: High-speed atomic force microscopy combined with mutational analyses clearly showed that FlhAL is required for highly corporative ring formation on mica surface (Terahara et al. Sci. Adv. 2018) and FlhAL of a closed form of FlhAC does not efficiently bind to the nearest FlhAC subunit to promote the nonameric ring assembly (Inoue et al. Structure 2019). The authors should cite these two important papers.
The Terahara reference was already cited, we have added a citation to the inoue reference
3. P6, Discussion, the 2nd paragraph: Although the linker region of FlhA/SctV may not be flexible in the absence of other factors, it has been shown that SctVC interacts with a highly conserved FHIPEP loop, which is located between transmembrane helices 4 and 5 of SctV Hartmann, N., and Büttner, D. Mol. Plant‐Microbe Interact. 2013). Consistently, the FHIPEP loop functionally communicates with FlhAC during H+-coupled protein export (Erhardt et al. Mol. Microbiol. 2017), FlhAC must get close to the cytoplasmic membrane through an interaction between FlhAC and the FHIPEP loop. To avoid misleading, the authors should tone down their statement in this paragraph.

We note the referee’s comments and have reconsidered the text to ensure we do not rule out a rearrangement that brings the cytoplasmic domain closer to the TM regions. Although we do not have the particle distribution to allow structure solution of the transmembrane domain, the sideview of the 2D averages demonstrates that the majority of the particles in this view have a set distance between the micelle and the cytoplasmic ring. If the linker were truly flexible we would only see defined structure for one of the two rings, while the other would appear as a smear in the “Z” axis. However, we cannot rule out that the secretion cycle, which involves energy input either from the protonmotive force or ATP hydrolysis, would trigger conformational change, and that the conformation observed here and in the published tomograms represent a relaxed state.

Reviewer #2: The work by Kuhlen et al. presents two single particle cryo-EM structures of the cytoplasmic domain of the major export apparatus component FlhA/SctV of bacterial type III secretion systems in their nonameric ring states. Both structures, one of flagella and one of virulence-associated injectisomes, were solved in the open state, a state that had been observed only in monomeric structures to date. The new nonameric structures also show that the linker region at the N-termini of the cytoplasmic domains of FlhA/SctV contributes to ring assembly through hydrophobic interactions. Both structures of the cytoplasmic domains were solved of full-length proteins, including their large N-terminal transmembrane domains. Even though the structures of the transmembrane domains could not be solved, the clear separation of both domains, spaced apart by about 30 Å, indicates that these domains remain at a fixed distance in the fully assembled type III secretion complexes and do not undergo a pumping motion towards the membrane and back to drive substrate translocation.
The paper is very brief and clearly written and illustrated and provides a a basis for developing new hypothesis on assembly and function of type III secretion sytems.

I do not have any major or minor points to raise.

We thank the referee for their consideration of our manuscript.

6. PLOS authors have the option to publish the peer review history of their article ([what does this mean?](https://journals.plos.org/plosone/s/editorial-and-peer-review-process#loc-peer-review-history)). If published, this will include your full peer review and any attached files.


If you choose “no”, your identity will remain anonymous but your review may still be made public.


**Do you want your identity to be public for this peer review?** For information about this choice, including consent withdrawal, please see our [Privacy Policy](https://www.plos.org/privacy-policy).

Reviewer #1: No

Reviewer #2: **Yes:** Samuel Wagner
