# Supplementary material for: Nonameric structures of the cytoplasmic domain of FlhA and SctV in the context of the full-length protein
Source: PLoS One. 2021 Jun 18;16(6):e0252800. doi: 10.1371/journal.pone.0252800 (PMC8213127; doi:10.1371/journal.pone.0252800)
Supplement: Peer review history

# Author comment: Author response to previous submission

**DOI:** 10.1371/journal.pone.0252800.r001

3 May 2021

Attachment Submitted filename: r2r_ed.docx Click here for additional data file.

# Editor report: Decision Letter 0

**DOI:** 10.1371/journal.pone.0252800.r002

**Authors:** Eric Cascales

24 May 2021

Nonameric structures of the cytoplasmic domain of FlhA and SctV in the context of the full-length protein.

PONE-D-21-14634

Dear Susan,

Thank you for submitting your revised manuscript and for addressing the reviewer's comments. I am pleased to accept your manuscript for publication. Please note that your manuscript will be formally accepted once it meets all outstanding technical requirements.

Within one week, you’ll receive an e-mail detailing the required amendments. When these have been addressed, you’ll receive a formal acceptance letter and your manuscript will be scheduled for publication.

An invoice for payment will follow shortly after the formal acceptance. To ensure an efficient process, please log into Editorial Manager at http://www.editorialmanager.com/pone/, click the 'Update My Information' link at the top of the page, and double check that your user information is up-to-date. If you have any billing related questions, please contact our Author Billing department directly at authorbilling@plos.org.

If your institution or institutions have a press office, please notify them about your upcoming paper to help maximize its impact. If they’ll be preparing press materials, please inform our press team as soon as possible -- no later than 48 hours after receiving the formal acceptance. Your manuscript will remain under strict press embargo until 2 pm Eastern Time on the date of publication. For more information, please contact onepress@plos.org.

Kind regards,

Eric

Eric Cascales

Academic Editor

PLOS ONE

Additional Editor Comments (optional):

Reviewers' comments:

# Editor report: Acceptance letter

**DOI:** 10.1371/journal.pone.0252800.r003

**Authors:** Eric Cascales

9 Jun 2021

PONE-D-21-14634 

Nonameric structures of the cytoplasmic domain of FlhA and SctV in the context of the full-length protein. 

Dear Dr. Lea:

I'm pleased to inform you that your manuscript has been deemed suitable for publication in PLOS ONE. Congratulations! Your manuscript is now with our production department. 

If your institution or institutions have a press office, please let them know about your upcoming paper now to help maximize its impact. If they'll be preparing press materials, please inform our press team within the next 48 hours. Your manuscript will remain under strict press embargo until 2 pm Eastern Time on the date of publication. For more information please contact onepress@plos.org.

If we can help with anything else, please email us at plosone@plos.org. 

Thank you for submitting your work to PLOS ONE and supporting open access. 

Kind regards, 

PLOS ONE Editorial Office Staff

on behalf of

Dr. Eric Cascales 

Academic Editor

PLOS ONE
